# Increased Availability of Selective Trace Elements Enhanced Anaerobic Benzoate Oxidation in *Geotalea daltonii*

**DOI:** 10.3390/microorganisms14040776

**Published:** 2026-03-29

**Authors:** Christina M. Kiessling, Cayden Samuels, Mary Arko, Xinyan Li, Kuk-Jeong Chin

**Affiliations:** Department of Biology, Georgia State University, Atlanta, GA 30303, USA; chrissi.kiessling@gmail.com (C.M.K.); csamuels6@student.gsu.edu (C.S.); arkomary92@gmail.com (M.A.); xinyanli985@gmail.com (X.L.)

**Keywords:** *Geotalea daltonii*, benzoate, anaerobic degradation, trace elements, ECF transporter

## Abstract

Anaerobic biodegradation of aromatic contaminants is constrained by unfavorable thermodynamics in the absence of oxygen and high activation energy required for aromatic ring-cleavage. Thus, identifying factors that enhance anaerobic aromatic degradation by microorganisms such as the *Geotalea daltonii* strain FRC-32 is crucial. Trace elements (TEs) function as rate-limiting cofactors for anaerobic carbon catabolism enzymes. Cobalt, molybdenum, selenite, and tungsten amendments stimulated *G. daltonii* growth on benzoate and anaerobic benzoate oxidation. To elucidate mechanisms of cobalt amendments in *G. daltonii*, we characterized a putative cobalt-specific energy-coupling factor (ECF) transporter CbiMNQO. The *cbiMNQO* genes form an operon and were upregulated under cobalt limitation, indicating a role in cobalt homeostasis. In silico structural predictions of CbiMNQO, ligand binding predictions of CbiMN, and alignment to known cobalt transporters suggested that CbiMNQO facilitates cobalt transport in *G. daltonii*. Structural and ligand binding predictions of BamB and BamF, and transcript-level analyses indicated that *bamB* and *bamF*, encoding molybdenum- and selenite–tungsten-dependent benzoyl-CoA reductase-subunits, modulate TE-dependent anaerobic benzoate degradation. Regulation of *bamB* and *bamF* in response to TE amendments corresponded with enhanced anaerobic benzoate oxidation, indicating stimulated benzoate dearomatization. Collectively, our findings demonstrated that TE amendments enhance anaerobic aromatic metabolism in *G. daltonii* and may contribute to anaerobic bioremediation.

## 1. Introduction

The accumulation of harmful contaminants in anaerobic environments, particularly petroleum-derived aromatic compounds, poses major challenges for bioremediation. In anaerobic environments, microorganisms must utilize alternative terminal electron acceptors (TEAs) other than oxygen, but oxygen is the TEA with the highest reductive potential. Using alternative TEAs thus reduces the thermodynamic and energetic redox potential of any carbon source that can be utilized [1,2,3,4]. Anaerobic metabolism leads to slower microbial growth and carbon source oxidation rates compared to aerobic metabolism [5,6]. Furthermore, aromatic compounds pose thermodynamic challenges for anaerobic biodegradation: aromatic compounds are not an energetically favorable carbon source due to their stable pi bond ring structures which require high activation energies to cleave the aromatic ring [7]. These challenges highlight the need to identify factors that promote anaerobic microbial growth and enhance anaerobic oxidation of aromatic contaminants [8,9,10].

The availability of trace elements (TEs) has been recognized as a possible factor enhancing anaerobic microbial activity [11,12]. TEs are known to play an essential role in anaerobic microbial metabolism because microorganisms utilize distinct metabolic pathways that are contingent upon the availability and enzymatic integrity of metalloenzymes [13,14]. TEs function as cofactors for many metalloenzymes including DNA and RNA polymerases, peroxidases, or dehydrogenases [15,16], DNA replication or repair [17], and transcription regulation [18,19]. Several studies have described the effects of cobalt, molybdenum, selenite, and tungstate on anaerobic growth and the anaerobic biodegradation of environmental contaminants. Florencio et al. [15] determined that cobalt supplementation stimulated microbial methanogenesis by enabling synthesis of corrinoid enzymes involved in methyl-coenzyme M formation, the direct precursor of methane. Wischgoll et al. [20] and Peters et al. [21] reported that selenite and molybdenum are essential for the enzymatic activation of anaerobic benzoyl-CoA metabolism in *Geobacter metallireducens* and *Desulfococcus multivorans*, respectively. Furthermore, multiple studies have confirmed the critical role of TE transport in microbial metabolism [10,22]. Particularly, cobalt acquisition and transport via energy-coupling factor (ECF) transporter were reported to be a key factor in linking extracellular cobalt availability to microbial metabolism [23,24,25]. ECF transporters belong to ATP-binding cassette transporters and mediate the uptake of micronutrients such as metal-cofactors in microorganisms [24].

Accordingly, TE availability is a potential lever for enhancing anaerobic aromatic degradation by microorganisms such as *G. daltonii*. Our investigation of the TEs cobalt, molybdenum, selenite, and tungstate was predicated on their reported roles in metalloenzymes that govern anaerobic aromatic oxidation—molybdenum, tungsten, and selenite were reported to mediate anaerobic aromatic ring reduction [20,21,26,27,28,29], while cobalt was reported to be essential for anaerobic carbon source metabolism [15,27,28].

We hypothesized that amendment of selective TEs, cobalt, molybdenum, selenite, and tungstate, will enhance anaerobic growth and anaerobic oxidation of aromatic compounds such as benzoate by *G. daltonii* FRC-32. In this study we aimed (1) to investigate the effect of cobalt, molybdenum, selenite and tungsten amendments on anaerobic growth and on anaerobic benzoate degradation in *G. daltonii*, (2) to identify and characterize the genetic and regulatory mechanisms of cobalt transport in *G. daltonii* by determining the organization, predicted function, and expression of the putative cobalt-specific ECF transporter encoded by *cbiMNQO* in response to cobalt amendments, and (3) to elucidate the effect of selenite, molybdenum, and tungsten amendments on modulation of anaerobic benzoate degradation via *bamB* and *bamF*, genes encoding molybdenum and selenite–tungsten-binding subunits of benzoyl-CoA reductase in *G. daltonii*.

By elucidating how trace element amendments modulate anaerobic growth and benzoate oxidation in *G. daltonii*, including their roles as essential cofactors for key enzymes and transport systems involved in anaerobic aromatic metabolism, this study establishes a framework to overcome thermodynamic and energetic constraints on anaerobic degradation of petroleum-derived aromatics. This framework provides a foundation for developing cost-effective and sustainable strategies to enhance the removal of aromatic contaminants from anoxic environments.

## 2. Materials and Methods

### 2.1. Culturing Methods

*G. daltonii* strain FRC-32 (DSM 22248; JCM 15807) [30] was cultured under strictly anaerobic conditions as previously described [31,32,33,34,35]. All cultures were performed in triplicate. Cultures were grown on 1 mM benzoate as a carbon source and electron donor, and 10 mM fumarate as the TEA. For growth studies with amendment of selective TEs, selenite was added in the form of sodium selenide (Na_2_Se), molybdenum was added in the form of sodium molybdate (Na_2_MoO_4_), cobalt was added in the form of cobalt chloride (CoCl_2_), and tungsten was added in the form of sodium tungsten (Na_2_WO_4_). The specific concentrations of the selective TEs which were added into the cultures are listed in Table 1. TE concentrations, herein referred to as “control” or “1×”, describe the concentrations of the TEs contained in the freshwater minimal medium used in this study. Trace element solution SL-10 (DSMZ 320) was prepared as described by the German Collection of Microorganisms and Cell Cultures (DSMZ, Braunschweig, Germany) [36]. TE concentrations, herein referred to as “no cobalt”, describe experimental conditions in which above-described bicarbonate-buffered freshwater minimal medium was prepared without cobalt chloride (CoCl_2_) in the trace element solution SL-10 (DSMZ 320). TE amendments were chosen as multiples (2.5× and 10×) of their respective 1× concentration in the freshwater minimal medium to explore the effect of TE amendment. These concentrations were selected based on previously reported ranges that stimulated anaerobic growth and substrate degradation in related microorganisms without causing toxic effects [15,26,27,28,37].

### 2.2. Total RNA Extraction

Total RNA was extracted from *G. daltonii* cultures as previously described [31,35,38,39]. Cells were disrupted for 1 min at 2500 rpm with a bead beater. RNA was subjected to TURBO DNase treatment (Life Technologies, Grand Island, NY, USA) which was confirmed via PCR and agarose gel electrophoresis. RNA concentration and purity were assessed using a NanoDrop 2000 UV-Vis Spectrophotometer (Thermo Fisher Scientific, Wilmington, DE, USA). All RNA samples were extracted from biological duplicate *G. daltonii* cultures.

### 2.3. Primer Design

The primers used in this study are listed in Table S1 and were synthesized by Integrated DNA Technologies (IDT) (San Jose, CA, USA). All primers except RACE-U-F and RACE-UT-F [31] were designed specifically for this study based on the full genome sequence of *G. daltonii* using the IDT PrimerQuest Tool (version 2.2.3) [40] and manually screened according to the primer selection guidelines [41]. Primers were tested for primer dimer and hairpin formation, and validity was confirmed using IDT OligoAnalyzer Tool (version 2.2.3) [40].

### 2.4. Reverse Transcription-Polymerase Chain Reaction (RT-PCR)

Synthesis of cDNA was performed using reverse primers (Table S1), 0.5 μg RNA, dNTP mix, RNase inhibitor and RevertAid RT reverse transcriptase (Thermo Fisher Scientific, Waltham, MA, USA) incubated at 42 °C for 60 min followed by enzyme inactivation at 70 °C for 10 min. cDNA products were verified by PCR amplification and agarose gel electrophoresis prior to downstream use.

### 2.5. Quantitative Real-Time Reverse Transcription PCR (qRT-PCR)

A dilution series of purified RT-PCR amplicons obtained with gene-specific primers were used as calibration standards as described previously [35,38,39]. All reactions were performed using SYBR Green PCR Master Mix (Life Technologies-Applied Biosystems, Grand Island, NY, USA) and 20 pmol of each primer pair. The temperature profile was composed of an initial activation step at 50 °C for 5 min and denaturation at 98 °C for 40 s, followed by 40 cycles of denaturation at 98 °C for 40 s, annealing at the primer-specific temperature for 32 s, and elongation at 65 °C for 32 s. Quantitative analysis was performed by the Applied Biosystems 7500 Real-Time PCR system (Life Technologies, Carlsbad, CA, USA) with 7500 Real-Time PCR System Sequence Detection Software (Version 2.0.6). PCR product size and specificity were confirmed via agarose gel electrophoresis and Sanger sequencing, respectively.

### 2.6. Rapid Amplification of 5′ cDNA Ends (5′ RACE)

RNA was extracted as previously described, treated with 0.1 M DTT, and incubated at 42 °C to eliminate secondary structure. cDNA was purified by incubation with 1 M NaOH at 65 °C for 20 min and GeneJet PCR Purification kit (Thermo Scientific, Pittsburgh, PA, USA,). DNA was tailed by incubation with Terminal Transferase (New England Biolabs, Ipswich, MA, USA). PCR was performed using Race UT-F primer for 43 cycles with 1 min of annealing at 54 °C, and 30 s of extension. This step was repeated, replacing the RaceUT-F primer with RaceU-F primer, and for the gene-specific reverse primer. PCR products were purified by GeneJet PCR Purification kit and sequenced via MinION (Oxford Nanopore Technologies, Oxford, UK) with the Rapid Barcoding Kit 96 V14.

### 2.7. Genetic Organization Analysis via Sequence-Overlap PCR (SO-PCR)

SO-PCR was performed as previously described [31,35,39] to sequence polycistronic mRNA transcripts. cDNA was synthesized as described above and PCR amplified using cDNA and respective SO-PCR reverse and forward primer pairs (Table S1). Amplicons were visualized by agarose gel electrophoresis and sequenced via Sanger sequencing. Sequences were aligned and concatenated via Clustal Omega (version 1.2.4) [42].

### 2.8. Identification of Anaerobic Benzoate Oxidation Genes and Cobalt Transport Genes

Genes were identified in the genome of *G. daltonii* (GenBank accession number NC_011979) by using the Basic Local Alignment Search Tool (BLAST version 2.17.0) for pairwise alignment to the genes of *G. metallireducens* (GenBank accession number NC_269799) [43,44].

### 2.9. Protein Domain and Functional Site Prediction

To predict functional residues and sites, amino acid sequences were submitted to ScanProsite [45]. ScanProsite utilizes the PROSITE database which contains a plethora of amino acid signatures. Once an amino acid sequence is submitted, a manually derived residue-based alignment may recognize known signatures, based on the PROSITE signature database [46]. To predict functional domains, amino acid sequences were submitted to Simple Modular Architecture Research Tool (SMART) [47,48] which identified domains and analyzed domain architectures based on residue-based alignment with their database.

### 2.10. In Silico Protein–Ligand Binding Affinity Prediction of BamB

To predict the protein–ligand binding affinity of the putative reductive dearomatization enzyme BamB for selective trace elements, AutoDock/Vina (version 1.2.x) was used [49,50]. Both ligand and protein were uploaded in pdbqt format, and a rectangular box was placed over the predicted binding site of the tested protein to precisely define the putative binding site. During the docking run, the interaction energy between each ligand atom and the receptor residue of the protein was calculated. Protein–ligand binding affinity predictions were represented as numerical values in kcal/mol: the lower the value, the higher the binding affinity [51]. Root mean square deviation (RMSD) values were calculated for each protein–ligand pose relative to the corresponding reference ligand structure. To graph and statistically differentiate the predicted binding affinities of the different tested substrates to BamB, a RMSD value difference of 2 Angstrom compared to the top prediction was used as a cut off. Docking predictions with RMSD values of below 2 Angstrom are generally considered unsuccessful [52].

### 2.11. Statistical Analysis

Unpaired two-tailed Student *t*-tests were performed for statistical analysis at a probability level of *p* < 0.05. For cell density measurement, the results represent the means ± standard errors of triplicate OD_600_ determinations for each sample obtained from triplicate cultures. For analysis of substrates and metabolites, the results represent the means ± standard errors of triplicate IC determinations for each sample obtained from triplicate cultures. For gene expression analysis, the results represent the means ± standard errors of the triplicate qRT-PCR determinations for each cDNA sample obtained from triplicate cultures.

## 3. Results and Discussion

### 3.1. Increased Cobalt Availability Enhanced Anaerobic Growth and Anaerobic Benzoate Oxidation in G. daltonii

**Figure 1:**
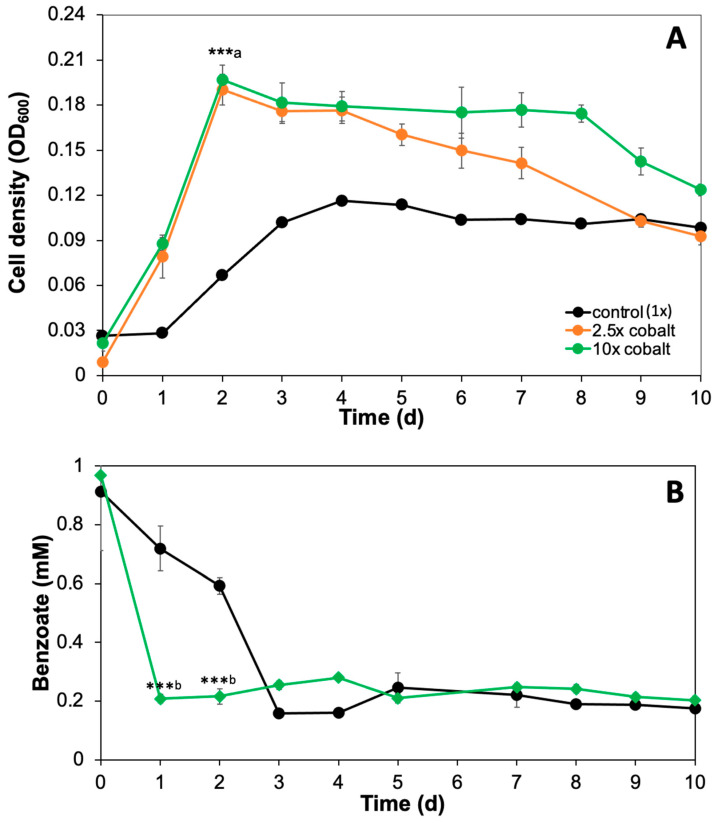
**Amendment of cobalt enhanced anaerobic growth of** ***G. daltonii*** **cultures on benzoate and anaerobic benzoate degradation in** ***G. daltonii***. (**A**) Anaerobic growth in cultures wih 2.5× and 10× cobalt amendments. (**B**) Anaerobic benzoate degradation in cultures with 2.5× and 10× cobalt amendments. The results represent the means ± standard errors of triplicate OD_600_ determinations or of triplicate IC determinations of each sample obtained from triplicate cultures (*** *p* > 0.0005, as determined by Student’s *t*-test). (a) Significant difference compared to cell density during growth on 1 mM benzoate without cobalt amendments is indicated by asterisks. (b) Significant difference compared to benzoate concentrations during growth on 1 mM benzoate with 10× cobalt is indicated by asterisks.

*G. daltonii* cultures were grown with and without cobalt amendment to determine the effect of cobalt availability on anaerobic growth and anaerobic benzoate oxidation by *G. daltonii.* Amendment of cobalt-enhanced anaerobic growth of *G. daltonii* cultures on benzoate as a carbon source compared to control cultures (1× cobalt) was performed, reaching cell densities of max OD_600_ of 0.190 (2.5× cobalt) and 0.197 (10× cobalt) (Figure 1A). Cultures with 2.5× cobalt entered the decline phase earlier (day 4) than cultures with 10× cobalt (day 8), suggesting that 10× cobalt was more beneficial for anaerobic *G. daltonii* growth. Growth rates of *G. daltonii* cultures were determined for each condition (Figure S1) to investigate the unconstrained metabolic efficiency during the logarithmic growth phase in response to cobalt availability. Growth rates of cultures grown with cobalt amendments (2.5× and 10× cobalt) were significantly higher than those of control cultures (1× cobalt). The ability of *G. daltonii* to anaerobically oxidize benzoate was assessed in cultures grown with 1× and 10× cobalt by monitoring benzoate oxidation over time (Figure 1B). Benzoate oxidation in cultures grown with 10× cobalt was enhanced compared to control cultures (1× cobalt) (Figure 1B). Benzoate was not completely oxidized, similarly to what we reported in our previous studies [31,39], likely due to accumulation of metabolic by-products or pH changes affecting enzyme activity. These findings corresponded to previous studies that reported that increased cobalt availability enhanced both anaerobic microbial growth and anaerobic carbon source degradation. Florencio et al. [15] reported that cobalt amendments enhanced anaerobic degradation of methanol in granular sludge containing *Butyribacterium methylotrophicum*, *Acetobacterium* sp., and *Methanosarcina barkeri* obtained from an anaerobic wastewater treatment plant. Our previous study [27] demonstrated that increased cobalt availability facilitated anaerobic methane production by stimulating microbial activity. Linville et al. [37] reported that increased availability of cobalt enhanced anaerobic microbial digestion of sewage sludge containing anaerobic bacteria from the following phyla: *Firmicutes*, *Bacteroidetes*, *Proteobacteria* and *Spirochaetes*.

### 3.2. Identification and Characterization of a Putative Cobalt ECF Transporter in Geotalea daltonii

**Figure 2:**
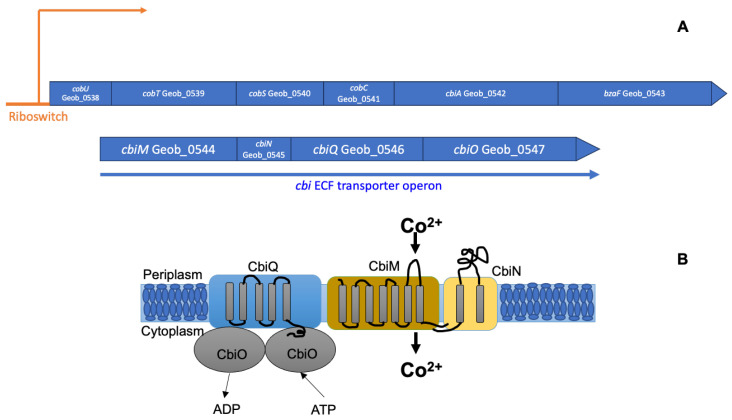
**Genetic organization of the** ***cbi*** **operon and proposed functional architecture of the ECF transporter CbiMNQO in** ***G. daltonii*****.** (**A**) The genes *cbiMNQO* are arranged in the *cbi* operon which is located immediately downstream of another putative operon encoding multiple genes that are proposed to be involved in vitamin B12 synthesis. A regulatory riboswitch is located upstream of both operons and is hypothesized to facilitate transcriptional control over both operons. (**B**) The Cbi system, a Group 1 ECF transporter, is known to facilitate substrate-specific transport of cobalt. This system is composed of four genes, *cbiMNQO*, which encode for the S module (*cbiMN*), the A module (*cbiO*), and the T module (*cbiQ*); the substrate-sensing and binding S module is energetically coupled to the AT module.

The enhancement of anaerobic growth and anaerobic benzoate oxidation in *G. daltonii*, observed under cobalt-amended conditions, indicated that cobalt availability modulated its metabolic capacity. Because cobalt functions as an essential cofactor for corrinoid-dependent enzymes and other metalloproteins involved in anaerobic carbon metabolism [22,53,54], increased rates of growth and benzoate oxidation (Figure S1) were hypothesized to lead to a higher intracellular demand for cobalt. At the same time, cobalt can be cytotoxic when accumulated in excess [28,55,56], requiring tightly regulated uptake systems to balance metabolic demand with metal homeostasis. Cobalt acquisition and transport may therefore represent a key factor in linking extracellular cobalt availability to enhanced anaerobic metabolism in *G. daltonii*. In silico genome analysis revealed the presence of adjacently located genes, putatively encoding a group 1 ECF-transporter Cbi in *G. daltonii* with the proposed function of cobalt transport (Figure 2) [44]. The Cbi transporter in *G. daltonii* was predicted to be encoded by the genes *cbiMNQO* (Figure 2A): *cbiMN* (Geob_0544 and Geob_0545) encode the substrate sensing and binding S-module. The S-module of metal transport systems, such as Cbi, has previously been reported to be encoded by two genes [57,58,59]. The gene *cbiQ* (Geob_0546) encodes the transmembrane T-module which is found at the center of the structure of ECF-transporters, building a scaffold to connect the S-module to the A-module [60]. The gene *cbiO* (Geob_0547) encodes the A-module that facilitates ATP hydrolysis [44] (Table S2). The A-module of ECF-transporters forms a dimer that functions very similarly to the well-studied ATPases of other ABC-transporters [23,59,61,62].

**Figure 3:**
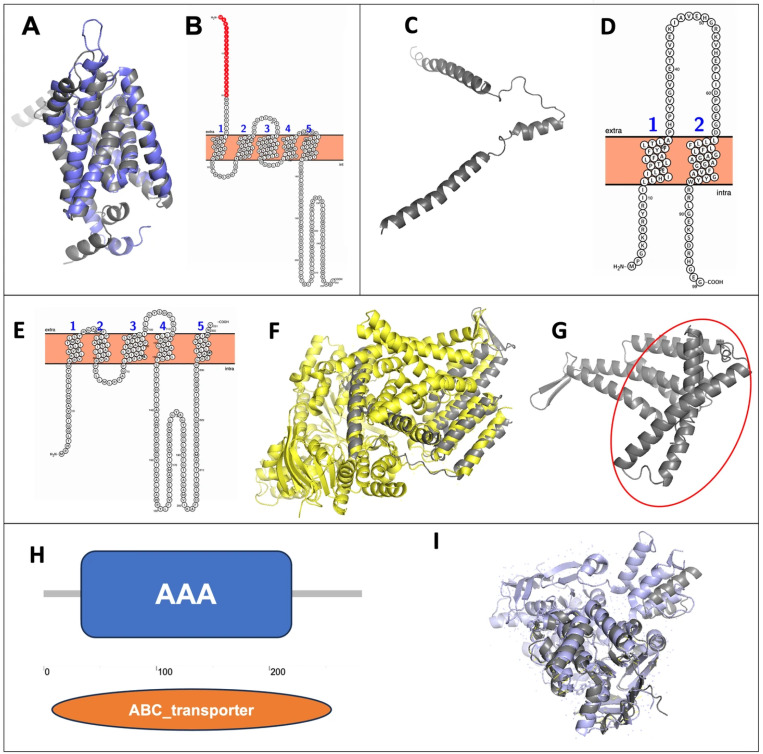
**Structure and function predictions for CbiM, CbiN, CbiQ, and CbiO.** (**A**) Structural alignment prediction of CbiM compared to *Rhodobacter capsulatus*’s CbiM (gray: *G. daltonii*; purple: *R. capsulatus*). (**B**) Protein topology prediction revealed that CbiM is membrane bound with an extracellular sensing domain at the C-terminal. Blue digits indicate the number of transmembrane regions. (**C**) Structure prediction of CbiM. (**D**) Protein topology prediction revealed that CbiN is membrane bound. Blue digits indicate the number of transmembrane regions. (**E**) Protein topology prediction revealed that CbiQ is membrane bound. Blue digits indicate the number of transmembrane regions. (**F**) Structural alignment prediction of CbiQ compared to *R. capsulatus*’ CbiQ (gray: *G. daltonii*; yellow: *R. capsulatus*). (**G**) Structure prediction of CbiQ with typical “X-shape” (shown inside the red circle). (**H**) In silico function prediction via ProSite revealed the presence of the ATPase domain (AAA) and the presence of the ATP-binding cassette, ABC transporter-type domain profile. (**I**) Structural alignment prediction of CbiO compared to *Caldanaerobacter subterraneus* subsp. *tengcongensis*’s CbiO (gray: *G. daltonii*; purple: *C. subterraneus*).

The structure of the S-module component CbiM of *G. daltonii* was predicted to have multiple transmembrane helices. S-modules of ECF transporters have been reported to share a conserved structure of transmembrane-spanning helices that form a barrel-shape with the N and C terminus of the protein extending into the cytoplasm or the extracellular space [63,64,65]. In silico structural alignment of *G. daltonii* CbiM with *Rhodobacter capsulatus* CbiM (PDB code 5X3X-2), reported by Bao et al. yielded an RMSD of 1.00 Å over 169 aligned residues, suggesting a high degree of conserved structural topology (Figure 3A,B) [62]. Bao et al. [62] reported the crystal structure of CbiM from *R. capsulatus* and showed, using in vitro substrate binding assays, that CbiM mediates substrate binding and release. In silico ligand binding predictions for *G. daltonii* CbiM indicated that it binds metal ions such as cobalt, supporting our hypothesis that CbiM functions in cobalt binding. In silico structure prediction for *G. daltonii* CbiN revealed three alpha-helices forming V-shaped transmembrane regions (Figure 3C,D), supporting our hypothesis that CbiN constitutes a component of the transmembrane S-module (Figure 2) [66]. The results of the in silico structural and functional predictions for CbiMN support our hypothesis that CbiMN functions as a substrate-sensing and binding domain for extracellular cobalt in *G. daltonii*. The structure of *G. daltonii’s* CbiQ was predicted to contain multiple transmembrane α-helices and cytoplasmic helices (Figure 3E), consistent with the functional prediction that *cbiQ* encodes a transmembrane protein (Figure 2) [62]. In silico structural alignment prediction for *G. daltonii* CbiQ with *Rhodobacter capsulatus* CbiQ yielded an RMSD of 2.36 Å over 166 aligned residues (Figure 3F), suggesting a moderately high degree of structural topology conservation. The typical X-shape [60] of Cbi transporter T-module cytoplasmic helices was identified in *G. daltonii* CbiQ (Figure 3F,G). This X-shaped coupling domain of the T-module was reported to be located on the cytoplasmic side of the cell membrane, where it binds to the A-module [60]. A conserved ATP-binding region [47,67] was identified in *G. daltonii* CbiO (Figure 3H). In silico structural alignment with *Rhodobacter capsulatus* CbiO yielded an RMSD of 1.05 Å over 209 aligned residues, suggesting a high degree of structural conservation (Figure 3I).

To refine functional predictions for the CbiMNQO complex, we examined the genomic context of the *cbiMNQO* genes in *G. daltonii*. We identified the following genes, which encode proteins proposed to facilitate vitamin B12 synthesis, located adjacent to the *cbiMNQO* genes in the genome of *G. daltonii*: *cobU* (bifunctional adenosylcobinamide kinase/adenosylcobinamide-phosphate guanylyl-transferase, Geob_0538), *cobT* (nicotinate-nucleotide-dimethyl-benzimidazolephosphoribosyl-transferase, Geob_0539), *cobS* (adenosylcobinamide-GDP ribazole-transferase, Geob_0540), *cobC* (alpha-ribazole phosphatase, Geob_0541), *cbiA* (cobyrinate a,c-diamide synthase, Geob_0542), and *bzaF* (5-hydroxybenzimidazole synthase, Geob_0543) (Figure 2 and Figure S2, Table S2) [43]. Additionally, a regulatory riboswitch was identified upstream of the *cbiMNQO* genes, which was predicted to bind vitamin B12 (as adenosyl-cobalamin) with a binding affinity of −23.43 kcal/mol and to function as transcriptional regulator in response to vitamin B12 (Figure 2) [68,69]. These findings supported our hypothesis that CbiMNQO facilitates cobalt transport in *G. daltonii* [70,71,72]. Rodionov et al. [73] reported that cobalt transporter genes were located adjacently to the genes that are involved in cobalt-dependent vitamin B12 synthesis in many prokaryotic genomes.

The predicted function of the CbiMNQO complex was further elucidated by analyzing the genetic arrangement of the *cbiMNQO* genes and whether these genes were expressed by a single promoter. In silico operon prediction indicated that the genes *cbiMNQO* in *G. daltonii* are arranged as a single operon. This arrangement was confirmed in vitro via SO-PCR (Table S3 and Figures S3–S5). Arrangement in one operon confirmed that Cbi in *G. daltonii* is a group 1 ECF-transporter. Group 1 ECF-transporters, particularly Cbi transport systems in other microorganisms, were previously reported to be organized as a single operon [23,24,62,74]. This genetic organization facilitates coordinated and simultaneous expression of the ECF-transporter genes. Furthermore, co-expression of *cbiMNQO* may allow *G. daltonii* to conserve resources: simultaneous expression of multiple genes necessary for cobalt transport could be facilitated (or prevented if necessary) at low energetic costs.

The promoter region of *cbiMNQO* was analyzed to identify regulatory elements and to understand regulation of *cbiMNQO*. The +1 transcription start site (+1 TSS) of the *cbi* promoter was mapped 53 nucleotides downstream of the translation initiation site of *cbiM* (Figure S6). Only one +1 TSS was identified, suggesting that transcription initiation is governed by a single promoter region. Identification of only one +1 TSS corresponded to our findings that the genes *cbiMNQO* are arranged in one operon (Table S3 and Figures S3–S5) and transcribed into one polycistronic mRNA transcript, confirming that the Cbi transporter in *G. daltonii* is a group 1 ECF-transporter.

**Figure 4:**
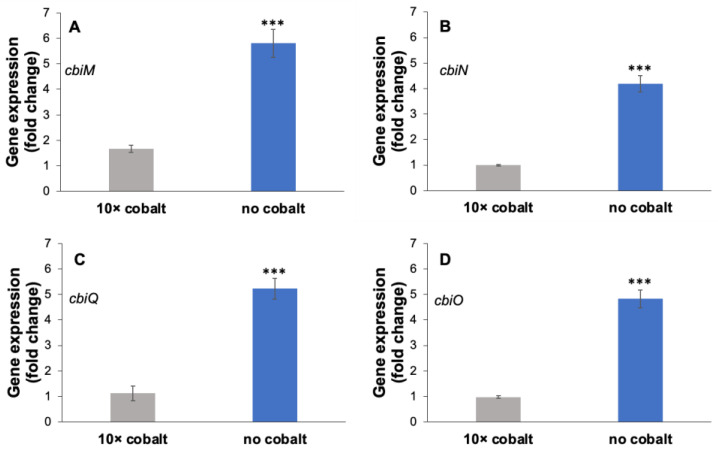
**Relative expression levels for** ***cbiMNQO*** **in** ***G. daltonii*** **cultures on benzoate with cobalt amendments.** (**A**) Expression levels for *cbiM*. (**B**) Expression levels for *cbiN*. (**C**) Expression levels for *cbiQ*. (**D**) Expression levels for *cbiO*. Transcript levels were normalized to transcript levels for housekeeping gene *recA*. Fold change was normalized to transcript levels for *cbiMNQO* in *G. daltonii* cultures on benzoate with 1× cobalt (control). The results represent the means ± standard errors of the triplicate qRT-PCR determinations of each cDNA sample obtained from triplicate cultures (*** *p* > 0.0005, as determined by Student’s *t*-test). Significant difference compared to expression of *cbiM*, *cbiN*, *cbiO*, or *cbiQ* in cultures grown with 10× cobalt is indicated by asterisks.

To assess the role of cobalt in regulating *cbiMNQO* expression, transcript levels were quantified in vitro under varying cobalt availability. Fold changes were calculated relative to *cbiMNQO* transcript levels in cultures grown on benzoate with 1× cobalt (control). Relative expression levels for *cbiM*, *cbiN*, *cbiQ*, or *cbiO* were significantly higher in *G. daltonii* cultures grown on benzoate during cobalt starvation (“no cobalt”) compared to ones in *G. daltonii* cultures on benzoate grown with 10× cobalt (by 4-fold, 3-fold, 4-fold, and 4-fold, respectively) (Figure 3). This demonstrated that expression of *cbiMNQO* was regulated in response to cobalt availability, particularly, that expression was upregulated during cobalt starvation (Figure 4). Our findings corresponded to the findings reported in several studies describing upregulation of metal-transporters during metal starvation in microorganisms. For example, Graf et al. [75] reported that multiple genes in *Anabaena* sp. PCC 7120, encoding proteins that facilitate uptake of cobalt or synthesis of vitamin B12 such as metal binding protein Alr4027 and cobalamin transporter protein BtuB, were upregulated in response to intracellular cobalt starvation. Martínez-Torró et al. [76] reported that many genes coding for predicted lipoproteins and ABC-transporters, such as MG_302, MG_303 and MG_304, were significantly upregulated during metal starvation in *Mycoplasma genitalium.* Relative expression levels for *cbiMNQO* remained unchanged in *G. daltonii* cultures grown with 10× cobalt compared to ones in control cultures which may prevent high, possibly toxic, intracellular accumulation of cobalt.

Our findings indicate that *G. daltonii* employed product sensing to regulate *cbiMNQO*: transcript levels responded to cobalt availability, and a vitamin B12-binding riboswitch is located upstream of the *cbi* and *cob* genes, which are predicted to facilitate vitamin B12 synthesis (Table S2, Figure S2). Product sensing is a regulatory mechanism which maintains intracellular cobalt levels by monitoring the product of cobalt metabolism, vitamin B12 [77]. Multiple studies have confirmed that vitamin B12-binding riboswitch regulate expression of *cbi genes* [73,77,78]. Furthermore, O’Brian [79] similarly reported that *Rhizobia* species regulate iron-dependent gene expression in response to iron-dependent processes, particularly, heme synthesis.

Co-regulation (similar fold change) of *cbiM*, *cbiN*, *cbiQ*, and *cbiO* corresponds to our previous findings that the *cbiMNQO* genes are arranged in an operon and transcribed into a single polycistronic mRNA transcript.

### 3.3. Increased Availability of Selenite, Tungsten, and Molybdenum-Enhanced Anaerobic Growth and Anaerobic Benzoate Oxidation in G. daltonii

**Figure 5:**
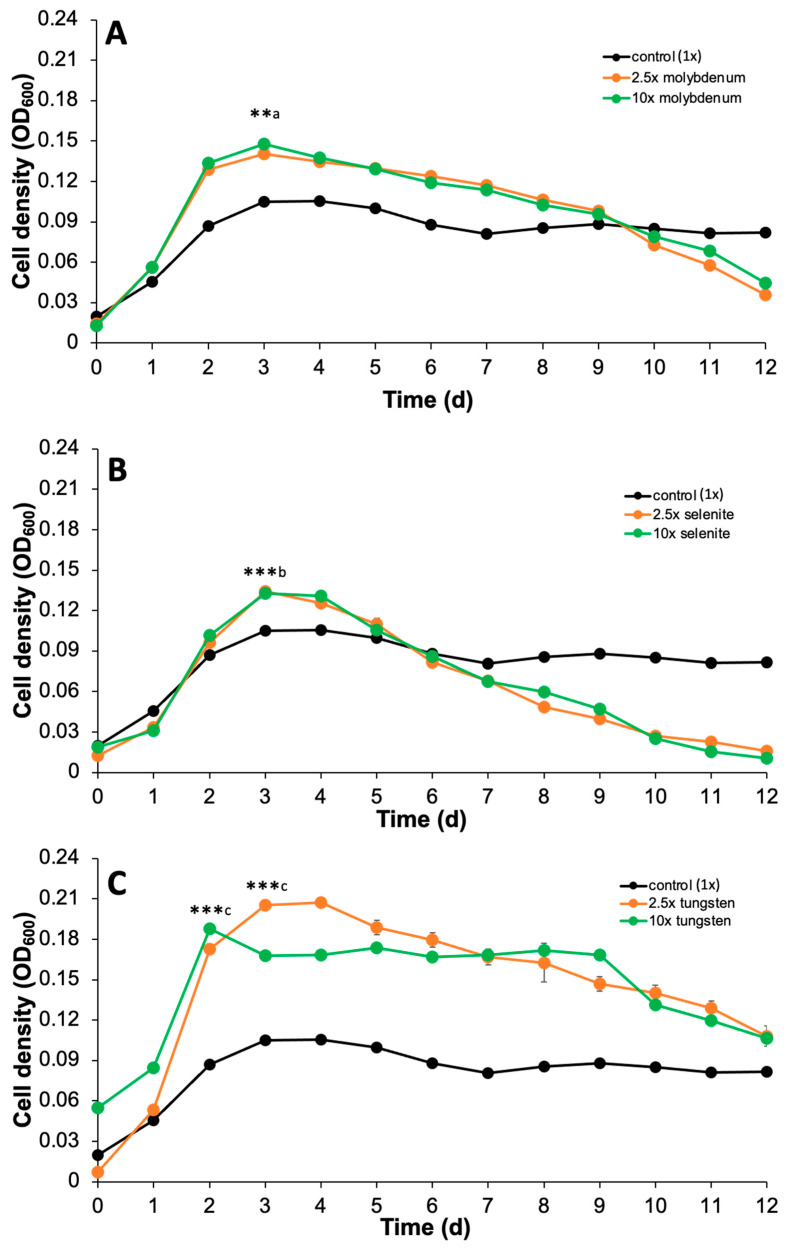
**Amendment of molybdenum, selenite and tungsten enhanced anaerobic growth of** ***G. daltonii*** **cultures with benzoate as carbon source.** (**A**) Anaerobic growth with molybdenum amendments. (**B**) Anaerobic growth with selenite amendments. (**C**) Anaerobc growth with tungsten amendments. The results represent the means ± standard errors of triplicate OD_600_ determinations of each sample obtained from triplicate cultures (*** *p* > 0.0005, ** *p* > 0.005 as determined by Student’s *t*-test). Significant difference compared to cell density during growth on 1 mM benzoate with (a) 1× molybdenum, (b) 1× selenite, or (c) 1× tungsten is indicated by asterisks.

The genes *bamBCDEFGHI*, hypothesized to facilitate aromatic ring activation during benzoate oxidation by functioning as benzoyl-CoA reductase (BCR), were identified in the genome of *G. daltonii* (Table S4). In silico ligand binding and functional predictions were made for BamBCDEFGHI in *G. daltonii*: BamB was predicted to function as a molybdenum and tungsten binding subunit that facilitates ligand binding, BamDE was predicted to function as an oxidoreductase that facilitates electron transfer, BamF was predicted to function as selenite binding electron transfer protein, and BamGHI was predicted to function as a NAD(P)+ binding electron output module (Table S4). Similarly, Carmona et al. [80] reported that BamBCDEFGHI function as BCR in aromatic ring reduction during anaerobic benzoate oxidation in *G. metallireducens*. In silico ligand binding prediction revealed that selenite, tungsten, and molybdenum function as cofactors of BamB and BamF, indicating that they play a role during anaerobic benzoate oxidation in *G. daltonii*. We investigated the effect of amendments of molybdenum, tungsten, and selenite on anaerobic growth and anaerobic benzoate oxidation in *G. daltonii*. Selective TE concentrations are listed in Table 1. Molybdenum amendments enhanced the growth of *G. daltonii* cultures grown on benzoate, reaching maximal cell densities of OD_600_ 0.141 (2.5× molybdenum) and 0.147 (10× molybdenum), respectively (Figure 5A). Maximal cell densities of cultures grown with 2.5× or 10× molybdenum were insignificantly different (Figure 5A). Growth rates of *G. daltonii* cultures were measured for each condition (Figure S8A) to determine the unconstrained metabolic efficiency during the logarithmic growth phase in response to TE availability. Growth rates of cultures grown with molybdenum amendments (2.5× and 10× molybdenum) were significantly higher than of control cultures (1× molybdenum). Selenite amendments enhanced growth of *G. daltonii* cultures grown on benzoate, reaching maximal cell densities of OD_600_ 0.136 (2.5× selenite) and 0.133 (10× selenite), respectively (Figure 5B). Maximal cell densities of cultures grown with 2.5× or 10× selenite were not significantly different (Figure 5B). Growth rates of *G. daltonii* cultures were determined for each condition (Figure S8B). Growth rates of cultures grown with selenite amendments (2.5× and 10× selenite) were significantly higher than of control cultures (1× selenite). Tungsten amendments enhanced growth of *G. daltonii* cultures grown on benzoate, reaching maximal cell densities of OD_600_ 0.188 (10× tungsten) and 0.207 (2.5× tungsten) (Figure 5C). Maximal cell densities in cultures grown with 2.5× tungsten were higher than with 10× tungsten (Figure 5C). Growth rates of *G. daltonii* cultures were determined for each condition (Figure S8C). Growth rates of cultures grown with tungsten amendments (2.5× and 10× tungsten) were significantly higher than control cultures (1× tungsten).

The growth characteristics of anaerobic *G. daltonii* cultures on benzoate demonstrated that selenite, tungsten, and molybdenum amendments enhanced anaerobic growth (Figure 5). Cultures with selenite or molybdenum amendments entered death phase faster than control cultures (1×) (Figure 5A,B) which could be explained by multiple factors such as rapid depletion of nutrients or accumulation of metabolic byproducts and waste products. Cell densities in benzoate-grown cultures with tungsten amendments were higher during the logarithmic phase and declined more slowly than in cultures with selenite and molybdenum amendments (Figure 5), indicating that tungsten amendments were more beneficial for growth of *G. daltonii* cultures on benzoate. Our finding that cell densities in cultures with 10× selenite or 10× molybdenum were not higher than those in cultures with 2.5× selenite or 2.5× molybdenum, respectively (Figure 5A,B), indicates that 2.5× TE availabilities may be sufficient for BamB and BamF to reach maximum catalytic velocity [81]. The lower maximal cell densities in cultures with 10× tungsten than with 2.5× tungsten suggest that 10× tungsten availability led to oversaturation, possibly causing an inhibitory effect on BamB and BamF. High concentrations of metal cofactors can inhibit enzyme function or even cause loss of enzyme activity [82], which would decelerate anaerobic benzoate oxidation and thereby slow microbial growth. Ligand binding prediction revealed that BamB requires both molybdenum and tungsten to form the catalytic center for the crucial initial electron transfer step in the reductive dearomatization of benzoyl-CoA (Table S2) [29]. Furthermore, in silico ligand binding affinity prediction suggested a higher binding affinity of BamB1 and BamB3 for tungsten (in its active form tungstopterin) than for molybdenum (in its active form of molybdopterin) (Figure S7). According to Michaeles-Menten enzyme kinetics, higher substrate affinity corresponds to a lower *Km*, the concentration required for half-maximal velocity [83,84,85]. These principles support our hypothesis that a lower concentration of tungsten, 2.5×, was sufficient for BamB1/BamB3 binding saturation in *G. daltonii*. 10× tungsten availability may cause oversaturation and inhibit BamB function [85], thereby slowing anaerobic benzoate oxidation and *G. daltonii* growth.

**Figure 6:**
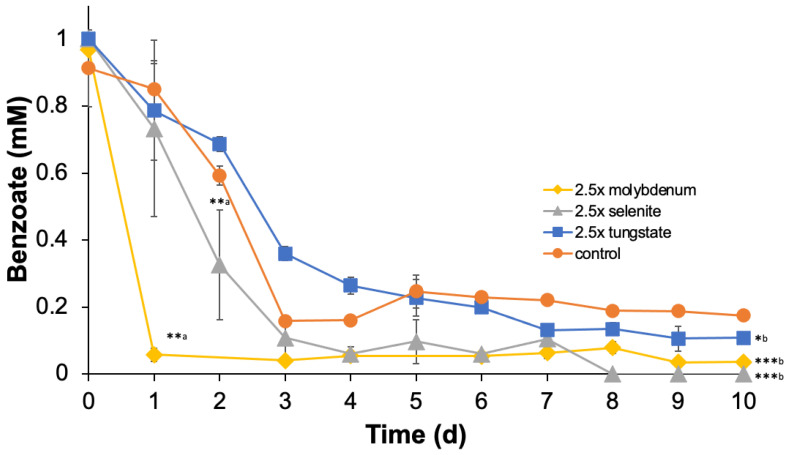
**Amendment of molybdenum, selenite, and tungsten enhanced anaerobic benzoate degradation in** ***G. daltonii.*** The results represent the means ± standard errors of triplicate IC determinations of each sample obtained from triplicate cultures (*** *p* > 0.0005, ** *p* > 0.005, * *p* > 0.5, as determined by *t*-test). Significant difference compared to benzoate concentrations during growth on 1 mM benzoate without trace element amendments during the logarithmic growth phases shown in (a) or during the decline phases shown in (b) is indicated by asterisks.

The effect of TE amendments on anaerobic benzoate oxidation in *G. daltonii* was assessed by measuring benzoate loss in cultures grown on benzoate with 2.5× selenite, 2.5× molybdenum, or 2.5× tungsten (Figure 6). In cultures grown with 2.5× molybdenum, benzoate was oxidized faster than in control cultures (1×), demonstrating that increased molybdenum availability enhanced anaerobic benzoate oxidation in *G. daltonii* (Figure 6). In cultures grown on benzoate with 2.5× selenite, benzoate was oxidized faster than in control cultures (1×), demonstrating that amended selenite availability enhanced anaerobic benzoate oxidation in *G. daltonii* (Figure 6). In cultures grown on benzoate with 2.5× tungsten, benzoate was oxidized faster than in control cultures (1×), although not significantly (Figure 6). At the end of the incubation period, benzoate concentrations were lower in cultures grown with 2.5× selenite, 2.5× molybdenum, or 2.5× tungsten than in control cultures (1×), demonstrating that increased TE availability facilitated more effective anaerobic benzoate oxidation in *G. daltonii*.

In summary, our findings support the hypothesis that selenite, molybdenum, and tungsten contribute to anaerobic benzoate oxidation by *G. daltonii*, likely functioning as cofactors for BamB and BamF, which facilitate aromatic ring reduction of benzoyl-CoA during benzoate oxidation. In our previous study [27], we demonstrated that increased availability of cobalt, copper, and molybdenum facilitated anaerobic methane production by stimulating microbial activity. These results align with the findings of Linville et al. [37], who reported that elevated nickel or cobalt enhanced anaerobic digestion of sewage sludge, and with Chakrabarti [26], who showed that selenite or molybdenum enhanced microbial nitrate reduction in anaerobic wastewater.

### 3.4. Differential Regulation of Anaerobic Reductive Dearomatization Genes bamB and bamF in Response to Amendment of Selenite, Molybdenum and Tungsten

**Figure 7:**
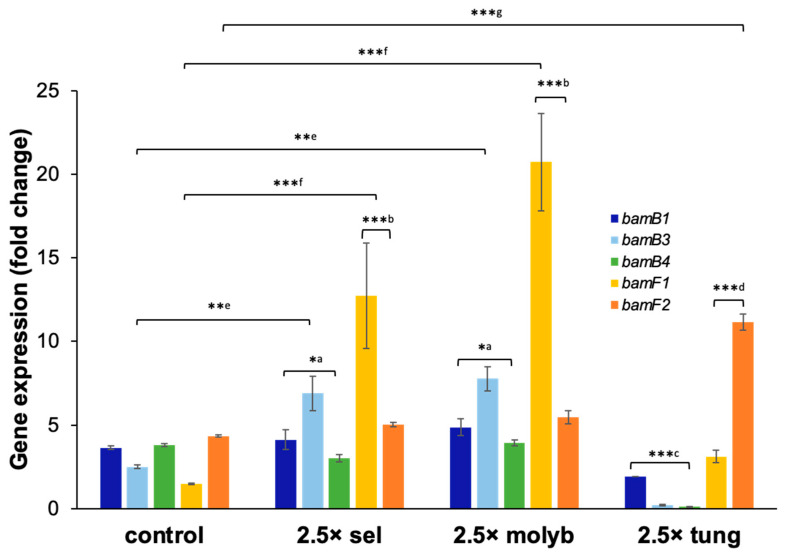
**Relative expression levels for putative benzoyl-CoA reductase genes** ***bamB1*****,** ***bamB3*****,** ***bamB4*****,** ***bamF1*** **and** ***bamF2*** **in** ***G. daltonii*** **cultures on benzoate with molybdenum, selenite and tungsten amendments.** Transcript levels were normalized to transcript levels for housekeeping gene *recA*. Fold change was normalized to transcript levels for *cbiMNQO* in *G. daltonii* cultures on benzoate with 1× molybdenum (control), 1× selenite (control), and 1× tungsten (control). The results represent the means ± standard errors of the triplicate qRT-PCR determinations of each cDNA sample obtained from triplicate cultures (*** *p* > 0.0005, ** *p* > 0.005, * *p* > 0.05 as determined by Student’s *t*-test). Significant difference compared to expression of (a) *bamB1* and *bamB4*, (b) *bamF2*, (c) *bamB3* and *bamB4*, (d) *bamF1*, (e) *bamB3* (control), (f) *bamF1* (control), or (g) *bamF2* (control) is indicated by asterisks.

To elucidate the role of selenite, molybdenum, and tungsten in modulation of reductive dearomatization genes *bamB* and *bamF* in *G. daltonii*, in vitro expression analysis was performed. Specifically, the response of the *bamB* homologs (*bamB1*, *bamB3*, and *bamB4*) and of the *bamF* homologs (*bamF1* and *bamF2*) to varying availability of these TEs were tested (Figure 7). Relative expression levels for *bamB1* did not increase in response to any of the tested selective TE amendments but decreased (by 1.5-fold) in cultures grown with 2.5× tungsten compared to control cultures (1×). Relative expression levels for *bamB3* increased in cultures grown with 2.5× selenite or 2.5× molybdenum (by 4.5-fold and 5-fold, respectively) and decreased (by 2.5-fold) in cultures grown with 2.5× tungsten compared to control cultures (1×). Relative expression levels for *bamB4* did not increase in response to any of the selective TE amendments but decreased (by 3-fold) in cultures grown with 2.5× tungsten compared to control cultures (1×).

Relative expression levels for *bamF1* increased in cultures grown with 2.5× selenite, 2.5× molybdenum, or 2.5× tungsten by 11-fold, 19-fold, and 1.5-fold, respectively, compared to control cultures (1×). Relative expression levels for *bamF2* did not increase in cultures grown with 2.5× selenite or 2.5× molybdenum but increased (by 8-fold) in cultures with 2.5× tungsten compared to control cultures (1×).

Our findings showed that only one *bamB* or *bamF* homolog was upregulated in response to amendment of molybdenum, selenite or tungsten. This selective expression suggests differential regulation that is energetically favorable for microorganisms: energy is conserved by expressing only the homolog that encodes the protein that is necessary under specific nutrition conditions [86]. Our previous study [31] demonstrated that the peripheral pathways that facilitate benzene, toluene, naphthalene, and benzoate oxidation in *G. daltonii* converged into a central pathway via the intermediate benzoyl-CoA, which is metabolized by the BCR-enzyme complex BamBCDEFGHI via aromatic ring activation. Similar differential gene regulation of *bamB* and *bamF* homologs in *G. daltonii* may occur during anaerobic oxidation of these diverse carbon sources, though this requires experimental validation.

Furthermore, higher relative expression levels for *bamB* and *bamF* in response to amendment of molybdenum, selenite and tungsten corresponded to our findings of enhanced microbial growth and anaerobic benzoate oxidation. Upregulation of *bamB* or *bamF* may lead to increased protein levels of BamB and BamF which could increase the rate of anaerobic benzoyl-CoA reduction.

## 4. Conclusions

In this study, we demonstrated that cobalt, molybdenum, selenite, and tungsten amendments significantly enhanced both anaerobic growth and metabolic activity in *G. daltonii*, highlighting the importance of metal cofactors in anaerobic degradation of petroleum-derived aromatic compounds such as benzoate. These findings suggested that selective TE amendments alleviated metabolic constraints associated with anaerobic aromatic metabolism. This work further advanced the understanding of cobalt acquisition in *G. daltonii* through characterization of the putative cobalt-specific ECF transporter CbiMNQO. In silico structural and functional analyses supported the role of CbiMNQO in cobalt uptake, while transcriptional analyses revealed differential expression of *cbiMNQO* in response to cobalt amendments, consistent with regulated intracellular cobalt homeostasis. In addition, differential expression of *bamB* and *bamF* in response to molybdenum, selenite, and tungsten amendments implicated these TEs in the reductive dearomatization step of anaerobic benzoate oxidation and suggested TE-dependent regulation of benzoyl-CoA reductase subunits. These findings suggested that selective TE amendments could be leveraged to enhance anaerobic bioremediation of aromatic contaminants in anoxic environments. By identifying which TEs stimulate key enzymes and transport systems in *G. daltonii*, it may be possible to optimize microbial activity in sediments, groundwater, and other oxygen-limited systems to accelerate the breakdown of petroleum-derived pollutants.

Future studies should experimentally validate CbiMNQO to resolve the architecture of the substrate-binding modules CbiM and CbiN and clarify their roles in cobalt binding and transport. Transport assays with cells expressing truncated *cbi* operon variants would dissect subunit-specific contributions to cobalt uptake. Mapping the transcription start site and analyzing regulatory elements of the *bamBCDEFGHI* operon will improve understanding of transcriptional control of anaerobic aromatic metabolism. Examining interactions between TEs and BamB and BamF will clarify how molybdenum, selenite, and tungsten availability modulate benzoyl-CoA reduction in *G. daltonii* and may guide strategies to optimize TE availability for anaerobic bioremediation. Investigating whether *G. daltonii* whole-cell lysates from benzoate-oxidizing cultures with increased concentrations of TEs show benzoate loss would clarify the effect of TE availability on benzoyl-CoA reduction. Lastly, future studies may evaluate anaerobic benzoate degradation with TE amendments by a defined microbial consortia including *G. daltonii* which may provide further insight into how interspecies interactions influence anaerobic benzoate degradation under environmentally relevant conditions.

## Figures and Tables

**Table 1:** Selective trace element concentrations in anaerobic *G. daltonii* cultures.

	1× (Control)	2.5×	10×
**Cobalt**	1.46 μM	3.65 μM	14.6 μM
**Molybdenum**	148.79 nM	371.975 nM	1487.9 nM
**Selenite**	34.69 nM	86.725 nM	346.9 nM
**Tungsten**	0.0272 nM	0.0675 nM	0.272 nM

## Data Availability

The original contributions presented in this study are included in the article and Supplementary Material. Further inquiries can be directed to the corresponding author.

## Supplementary Materials

The following supporting information can be downloaded at: https://www.mdpi.com/article/10.3390/microorganisms14040776/s1, Table S1: primers used in this study; Table S2: Genes encoding proteins proposed to be involved in cobalt transport or cobalt-dependent vitamin B12 synthesis in *G. daltonii*; Table S3: Pairwise alignment scores of RT-PCR SOPCR-amplicons; Table S4: Genes encoding enzymes proposed to facilitate anaerobic reductive dearomatization in *G. daltonii*; Figure S1: Growth rates and benzoate degradation rates in log phase of anaerobic *G. daltonii* cultures on benzoate with cobalt amendments; Figure S2: Proposed pathway for anaerobic vitamin B12 synthesis in *G. daltonii;* Figure S3: Products of sequence- overlap RT-PCR; Figure S4: Pairwise alignments of RT-PCR SOPCR-amplicons; Figure S5: Sequence- overlap PCR arrangement; Figure S6: Identification of the +1 transcription start site (+1 TSS) of *cbiM*; Figure S7: In -silico protein-ligand binding affinity prediction of BamB1, BamB3, and BamB4 for molybdo-pterin and tungsto-pterin; Figure S8: Growth rates and benzoate degradation rates in log phase of anaerobic *G. daltonii* cultures with TE amendments.

## Abbreviations

The following abbreviations are used in this manuscript:

5′ RACE	Rapid amplification of 5′ cDNA ends
ECF	Electron coupling factor
IC	Ion chromatography
qRT-PCR	Quantitative reverse transcription PCR
RMSD	Root mean square deviation
SDS-PAGE	Sodium-dodecyl polyacrylamide gel electrophoresis
SOPCR	Sequence-overlap PCR
TE	Trace elements
TEA	Terminal electron acceptor
