# Supplementary material for: Increased Availability of Selective Trace Elements Enhanced Anaerobic Benzoate Oxidation in Geotalea daltonii
Source: Microorganisms. 2026 Mar 29;14(4):776. doi: 10.3390/microorganisms14040776 (PMC13119186; doi:10.3390/microorganisms14040776)
Supplement: Supplementary file 1 [file microorganisms-14-00776-s001.zip › microorganisms-4204473-supplementary.pdf]

Supplementary Materials

Increased availability of selective trace elements enhanced anaerobic benzoate oxidation in *Geotalea daltonii*

Christina M Kiessling, Cayden Samuels, Mary Arko, Xinyan Li, and Kuk-Jeong Chin

This file includes:

1. Supplemental Tables S1-S4
2. Supplemental Figures S1-S8

1. Supplemental tables

Table S1. Primers used in this study*

Primer	Target gene	Primer sequence	Amplicon length (bps)
cbiM-F	Geob_0544	5′-CCACCTATCTGACCACCT-3′	108
cbiM-R		5′-GGCAACTGGGTAGGGATGAA-3′	
cbiN-F	Geob_0545	5'-CACATCATCCTGTTGCCCA-3'	204
cbiN-R		5'-CACAAAACCTCCCGCAGC-3'	
cbiQ-F	Geob_0546	5′-CAGATACAGGTCGCTGAT-3′	187
cbiQ-R		5′-CAAAACGAAGCATCAGCAA-3′	
cbiO-F	Geob_0547	5'-GCCAGAAAAAAAGGGTCT-3'	132
cbiO-R		5'-GGTTCAGCCTGGTCAAAA-3'	
cbiX-F	Geob_0029	5'-ATGGAAACAGCGATTTTGA-3'	210
cbiX-R		5'-AAAATAGGGAAGAAGCAGGA-3'	
recA-F	Geob_1115	5'-GTTGACCGCTATCATCAGTA-3'	275
recA-R		5'-AAATGCCCTCGCCGTAGAGAA-3'	
bamB1-F	Geob_0212	5'-TCGCCAAGCCACAGGAGT-3'	209
bamB1-R		5'-CTCTTCGGTCCAGAAATCCTT-3'	
bamB3-F	Geob_0227	5'-AACCCGTCCTGTGGCGAT-3'	201
bamB3-R		5'-CTCTCGTACCTCTTCCATTTCC-3'	

bamB4-F	Geob_0228	5'-AACCGTGAAGAGGGTGCC-3'	194
bamB4-R		5'-T GCT GAC GGA GGT GCG GTT-3'	
bamF1-F	Geob_0216	5'-GGTCTCAACGCATCTGAA-3'	105
bamF1-R		5'-TTACACTTGAGCCAGGG-3'	
bamF2-F	Geob_0232	5'-CCAGATTCTCACGCTCCTG-3'	148
bamF2-R		5'-ATAGCGTTTCTCGGTTTCGTTG-3'	
5′RACE-*cbiM-F*	Geob_0544	5′-TGAAAAAAATCCTGTTAT-3′	variable**
5′RACE-*cbiM-nested-R*	Geob_0544	5′-TGAAGACAACAGCAGCCA-3′	219
5′RACE-*cbiM-R*	Geob_0544	5′-GCAGGGATGGGAACAGGT-3′	275
RaceUT-F*	Poly-C tail of 5'- RACE product	5'-CGCGAATTCCTCTTCTAGATGGGIIGGGI-IGGGIIG-3'	variable**
RaceU-F*	RaceUT	5'-CGCGAATTCCTCTTCTAGATGG-3'	variable**
SOPCR-*cbi*-1F		5′-GCAAAGCGTGAAAGAAAAATA-3′	674
SOPCR-*cbi*-1R		5′-GGCAACTGGGTAGGGATGAA-3′	
SOPCR-*cbi*-2F		5′-CCACCTATCTGACCACCT-3′	686
SOPCR-*cbi*-2R		5′-CAAAACGAAGCATCAGCAA-3′	
SOPCR-*cbi*-3F	Geob_0544 - Geob_0547	5′-CAGATACAGGTCGCTGAT-3′	693
SOPCR-*cbi*-3R		5′-TTCCAGATAACTCCCAAAAC-3′	
SOPCR-*cbi*-4F		5′-ACAGCCAGACCATCACCAC-3′	661
SOPCR-*cbi*-4R		5′-GGTTCAGCCTGGTCAAAA-3′	
SOPCR-*cbi*-5F		5′-GCCAGAAAAAAAGGGTCT-3′	540
SOPCR-*cbi*-5R		5′-GCTATCTGGGTGAATGGCT-3′	

*All primers except for RaceUT-F and RaceU-F were designed in this study (Bullows *et al*. 2024).

**It indicates variable amplicon lengths based on insert size.

Table S2. Genes encoding proteins proposed to be involved in cobalt transport or cobalt-dependent vitamin B12 synthesis in *G. daltonii*

Gene	Gene name	Gene product
Geob_0029	*cbiK or cbiX*	Sirohydrochlorin-chelatase
Geob_0030	*cobH* or *cbiC*	Precorrin-8X methylmutase
Geob_0031	*cbiD*	Cobalt-precorrin-5B (C(1))-methyltransferase
Geob_0032	*cobL* or *cbiE*	Bifunctional cobalt-precorrin-7 (C(5))-methyltransferase/cobalt-precorrin-6B(C(15))-methyltransferase
Geob_0033	*cobI*	Precorrin-2 C(20)-methyltransferase
Geob_0034	*cobM*	Precorrin-4 C(11)-methyltransferase
Geob_0036	*cobG*	Cobalt-precorrin 5A hydrolase
Geob_0038	*cobQ*	Cobyric acid synthase

Geob_0039	*cbiB*	Adenosylcobinamide-phosphate synthase
Geob_0040	*cobD*	Threonine-phosphate decarboxylase
Geob_0538	*cobU*	Bifunctional adenosylcobinamide kinase/adenosylcobinamide-phosphate guanylyl-transferase
Geob_0539	*cobT*	Nicotinate-nucleotide—dimethyl benzimidazole phosphoribosyl transferase
Geob_0540	*cobS*	Adenosylcobinamide-GDP ribazole transferase
Geob_0541	*cobC*	Alpha-ribazole phosphatase
Geob_0542	*cbiA*	Cobyrinate a,c-diamide synthase
Geob_0543	*bzaF*	5-hydroxybenzimidazole synthase
Geob_0544	*cbiM*	Energy-coupling factor ABC transporter permease
Geob_0545	*cbiN*	Energy-coupling factor ABC transporter permease 2
Geob_0546	*cbiQ*	Cobalt ABC transporter permease, inner membrane subunit
Geob_0547	*cbiO*	Cobalt ABC transporter ATPase
Geob_1298	*cobA*	Uroporphyrinogen-III C-methyltransferase
Geob_1302	*cysG*	Bifunctional precorrin-2 dehydrogenase/sirohydrochlorin ferrochelatase

Table S3. Pairwise alignment scores of RT-PCR SOPCR-amplicons

Samples	Identity (%) of overlapping regions	E value
SOPCR 1 and SOPCR 2	88	4×10^{-27}
SOPCR 2 and SOPCR 3	95	2×10^{-74}
SOPCR 3 and SOPCR 4	74	6×10^{-21}
SOPCR 4 and SOPCR 5	82	2×10^{-36}

Table S4. Genes encoding enzymes proposed to facilitate anaerobic reductive dearomatization in *G. daltonii*

Gene name	Gene	Gene product
bamB1[*a]	Geob_0212	Benzoyl-CoA reductase, bis-(molybdopterin)-oxotungsten-binding subunit
bamC1[*a]	Geob_0213	Benzoyl-CoA reductase, iron-sulfur cluster-binding subunit
bamD1[*a]	Geob_0214	Iron-sulfur cluster-binding oxidoreductase, CCG domain pair-containing, benzoyl-CoA reductase electron transfer protein
bamE1[*a]	Geob_0215	Polyferredoxin, benzoyl-CoA reductase electron transfer protein
bamF1[*a]	Geob_0216	Benzoyl-CoA reductase electron transfer protein, selenocysteine-containing
bamG1[*a]	Geob_0217	Benzoyl-CoA reductase electron transfer protein

bamH1[*a]	Geob_0218	Benzoyl-CoA reductase electron transfer protein
bamI1[*a]	Geob_0219	Iron-sulfur cluster-binding protein
bamB3	Geob_0227	Benzoyl-CoA reductase, bis-(molybdopterin)-oxotungsten-binding subunit
bamB4[*b]	Geob_0228	Benzoyl-CoA reductase, bis-(molybdopterin)-oxotungsten-binding subunit
bamC3[*b]	Geob_0229	Benzoyl-CoA reductase, iron-sulfur cluster-binding subunit
bamD2[*b]	Geob_0230	Iron-sulfur cluster-binding oxidoreductase, CCG domain pair-containing, benzoyl- CoA reductase electron transfer protein
bamE2	Geob_0231	Polyferredoxin, benzoyl-CoA reductase electron transfer protein
bamF2[*c]	Geob_0232	Benzoyl-CoA reductase electron transfer protein, selenocysteine-containing
bamG2[*c]	Geob_0233	Benzoyl-CoA reductase electron transfer protein
bamH2[*c]	Geob_0234	Benzoyl-CoA reductase electron transfer protein
bamI2	Geob_0235	Iron-sulfur cluster-binding protein

[*a] Genes are proposed to be arranged in an operon.
[*b] Genes are proposed to be arranged in an operon.
[*c] Genes are proposed to be arranged in an operon.

2. Supplemental figures

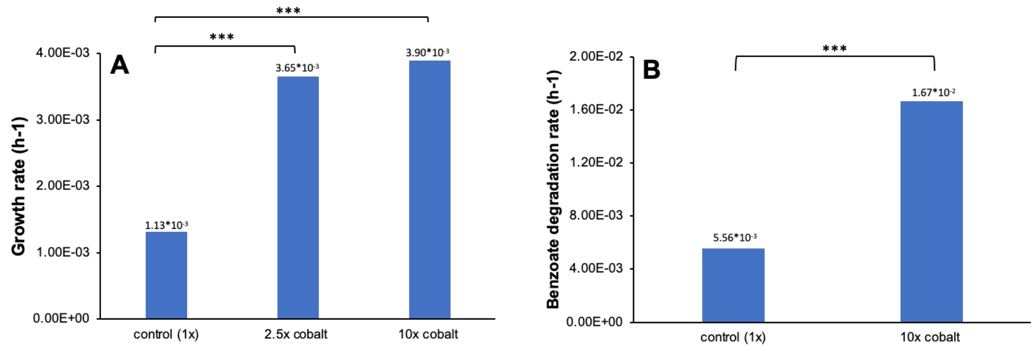

Figure S1. Growth rates and benzoate degradation rates in log phase of anaerobic *G. daltonii* cultures on benzoate with cobalt amendments. A: Growth rates were calculated based on the change of cell density over time during the logarithmic phase of each growth curve displayed in Fig. 1A. B: Benzoate degradation rates were calculated based on the change of benzoate concentrations over time during the logarithmic phase of each benzoate degradation curve displayed in Fig. 1B. The results represent the means ± standard errors of triplicate OD_{600} values or triplicate benzoate concentration values from triplicate cultures (****P* >0.0005; as determined by Student's *t*-test). A: Significant differences compared to growth rate of control cultures (1× cobalt) are indicated by asterisks. B: Significant difference compared to benzoate degradation rate of control cultures (1× cobalt) is indicated by asterisks.

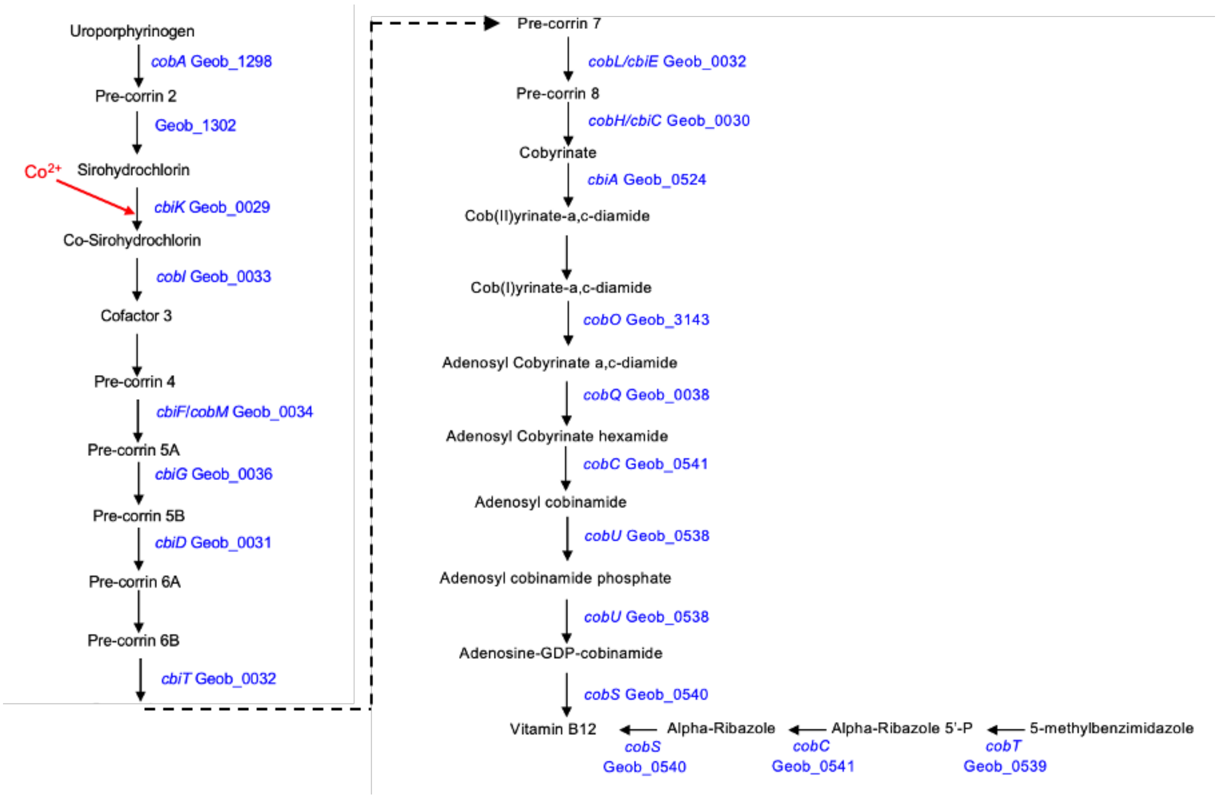

Figure S2. Proposed pathway for anaerobic vitamin B12 synthesis in *G. daltonii*. *In silico* analysis of the genome of *G. daltonii* revealed the presence of genes putatively involved in anaerobic vitamin B12 synthesis, supporting the hypothesize that *G. daltonii* has the genetic potential for anaerobic vitamin B12 biosynthesis. The arrow indicates insertion of Co^{2+} into sirohydrochlorin by the enzyme sirohydrochlorin cobaltochelatase, yielding cobalt-sirohydrochlorin.

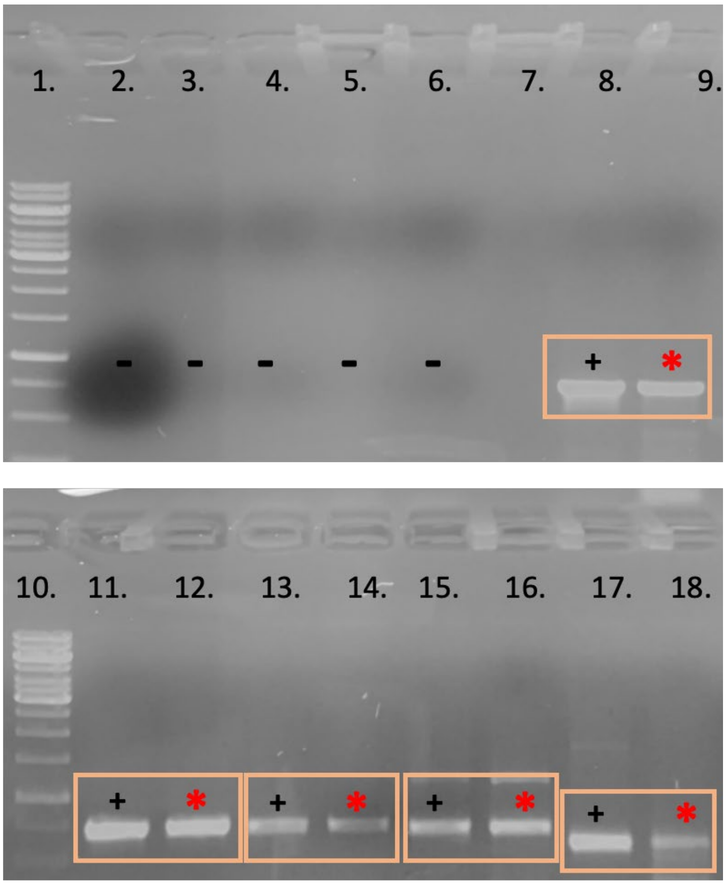

Figure S3. Products of sequence overlap RT-PCR. Each product represents a portion of the target operon. 1: 100 bp DNA ladder. 2: Negative control products of primers SOPCR-1. 3: Negative control products of primers SOPCR-2. 4: Negative control products of primers SOPCR-3. 5: Negative control products of primers SOPCR-4. 6: Negative control products of primers SOPCR-5.7: empty lane. 8: Positive control products of primers SOPCR-1 (674 bps). 9: RT-PCR products of primers SOPCR-1 (674 bps). 10: 100 bp DNA ladder. 11: Positive control products of primers SOPCR-2 (686 bps). 12: RT-PCR products of primers SOPCR-2 (686 bps). 13: Positive control products of primers SOPCR-3 (693 bps). 14: RT-PCR products of primers SOPCR-3 (693 bps). 15: Positive control products of primers SOPCR-4 (661 bps). 16: RT-PCR products of primers SOPCR-4 (661 bps). 17: Positive control products of primers SOPCR-5 (540 bps). 18: RT-PCR products of primers SOPCR-5 (540 bps).

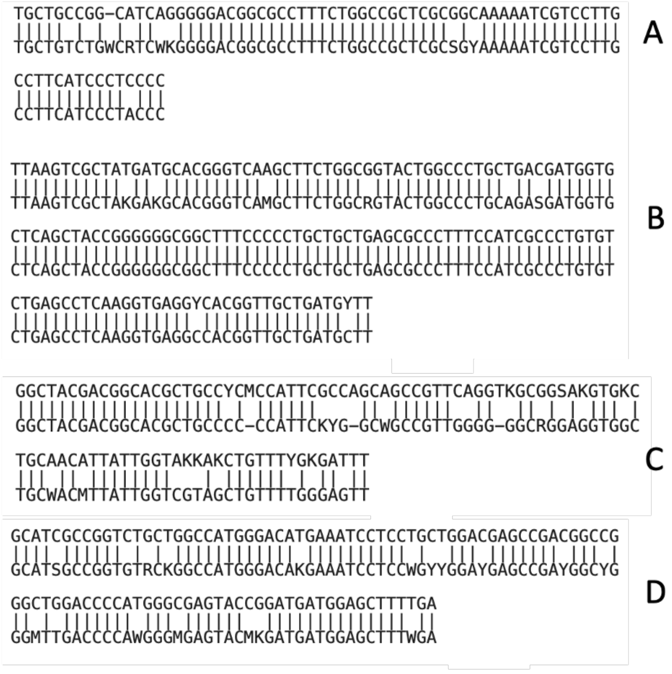

Figure S4. Pairwise alignments of RT-PCR SOPCR-amplicons. A. Alignment of SO-PCR amplicons 1 and 2. B. Alignment of SO-PCR amplicons 2 and 3. C. Alignment of SO-PCR amplicons 3 and 4. D. Alignment of SO-PCR amplicons 4 and 5.

Geob_0543 *cbiM*/Geob_0544 *cbiN*/Geob_0545 *cbiQ*/Geob_0546 *cbiO*/Geob_0547 Geob_0548
Overlapping regions

CCCGCGGGGCGGGAAAGCTGTTTGCCGGAGATTTGCGCGGAGACAAGATCTGAGGTGAAAAAATTCAGCCACGAATAGCACGAAAGTTCACGAATAAGAGCAAAGCGT
GAAAGAAAAATATGTGGTTGTGCTTCGGTTTGACTATCGTGTTAATTCGTGACATTCGTGGCTAAAAGATTTGAGATTCATAATGAAAAAAATCCTGTTATTTACAATTTTAA
TGCAGTTATTGCTGCCCCTGCCCATGCCATGCACATCAGTGAGGGGCTACTGCCACTGAACTGGGCGCTCTTCTGGTTCATGCGCGGCGCCTTTTGTCGCCCTTGGCGTGC
GTCAGCTGAATAACCTGGCAAAAACCGATCTGGCGATGAAACCGCTGTGGGGCTGATGGCTGCTGTTGTCTTCATCGTCTCCTGCATGCCCATCCCGGTCCCCACCGCCGG
CACCTGTTCCCATCCCTGCGGAACCGGCATTGCCGCTATCCTTGTCGGGCCTTTGGTCAGCGTTGTCATCAGTGCCGTGGCCTTCTGCTCCAGGCACTGTTTCTTGCCCATG
GCGGCCTTTCCACCCTGGGGGCGGACATTATTTCCATGGGGGTGGCCGGCCCTTTGCCGGCTGGCTAATCTTTCGGGGCCTGCGTAAAATCAGTGTCAACTTGGCCGTAGC
TGCTTTTTTCGGTGGTCTCTTTACGGACTGGGCCACCTATCTGACCACCTCCATGGAGCTTGCTGCCGGCATCAGGGGGACGGCGCCTTTCTGGCCGCTCGCGGCAAAAAT
CGTCCTTGCCTTCATCCCTACCCAGTTGCCGCTGGGCATTCTGGAAGGAGCCATGACCGCCGGCATGGTTACTCTGCTACAGCGCAAGCGCCCGGACCTGCTGGTGAAGA
TGCGGGTACTGAAGGAAAGCGAGGTCGTCCATGCCTGGAAAAAAGCGCAGATATAGAATAATCCTGTTGCACATCATCCTGTTGCCCACACTGCTCTTTGCCTTCTATTTC
CTTACCCTGGCGCCCCACCCCTATGTGGGGGTGGATGAGACGGTGGTCGAGAAAATTGCCGTGGAGCATGGCCGCAAGGTGCATGAACCCCTCATCGATCCGGGAGAGG
GAGATTTGCTGCTCTTTCTTTTTCTTGGAGCCGGTGCTGCGGGAGGTTTTGTGGCCGGTTATTACTGGCGCCGGTTGGGCGAGAAATCTGACCGGCATGGGGAAGGTTGAT
GCGTCATACCCTGCAACAGATACAGGTCGCTGATCATCCCTTAAGTCGCTATGATGCACGGGTCAAGCTTCTGGCGGTACTGGCCCTGCTGACGATGGTGCTCAGCTACCG
GGGGGCGGCTTTCCCCCTGCTGCTGAGCGCCCTTTCCATCGCCCTGTGTCTGAGCCTCAAGGTGAGGCCACGGTTGCTGATGCTTCGTTTTGCCCAGCCCCTTTTCTTTGC
CGCGGTGATCGTCCTGCTCAAGCTGTTCACTACCGGTGCAGTTCCCCTTTTTACCCTGTCTGTCTGGGGAATTAATCTTATCGGCTACAGTGACGGATTGGGCGAAGGGTT
GCTCATTGCCGGCCGGATCGTGGGGGCAGTTTCCCTGGTTGCCCTCCTCGGTTTTTCCACCTCCTTTACCGATCTGATTTCGGCCTTGGCCTGGTTTCGGGTGCCCCAGGG
GGTGATCGAGGTGGCCCTGTTTGCCTGGCGTTACCTGTTCGTTCTCTTTGAAGATGCCATGGTGATATACAACGCCCAGAAGAACCGTCTGGGTTATGCGGGTTATCCCCA
GGGGCTGCGTTCTTTCGGCACCCTTGCCGGCGCCATGGTCATAAAGGCCTTCGACAACAGCCAGACCATCACCACCGCCATGGTCCAGCGGGGCTACGACGGCACGCTGC
CCCCCATTCGCCAGCAGCCGTTCAGGGGGCGGGAGGTGGCTGCAACATTATTGGTCGTAGCTGTTTTGGGAGTTATCTGGAATGTTTAGCACCGACGTTCGTATTTCCGTG
AGCCTGGAGAGTTTCAAGTATCCCGATGGGACTGTGGCACTGTCTGATATTCATCTCGAGATCGCGCGGCGAGTTCTGCGGCATCCTCGGCGCCAACGGCTCGGGTAAGA
CCACACTGCTGAAGATCATGGATGGCCTGATCAAGGACTACCAGGGGCGGGTGCTTCTGGATGGGGAGGAGGTGCACCGGCTCCACCCCAGGGATATCTACCGCAAGATG
GGGCTCGTCTTCCAGAACCCCGATGATCAGCTCTTTGCCCATACCGTCTCTGAAGATGTGGCCTTCGGCCCCCGCAATATGGGCTGCGGCGAAAACGAGGTGAGAGGACG
GGTCGCGGATGCATTATTGGCGGTGGAAATGTCCGAATTCGGCGGCAAGGGTATTCACCATCTGAGCTATGGCCAGAAAAAAAGGGTCTGCATCGCCGGTCTGCTGGCCAT
GGGACATGAAATCCTCCTGCTGGACGAGCCGACGGCCGGGCTGGACCCCATGGGCGAGTACCGGATGATGGAGCTTTTGACCAGGCTGAACCGGGAAAATGGCGTGACC
ATAGTCATGGCTACCCACAGTGTTGACCTGGTGCCGATTTTTCTCCACCGGCTGCACATTCTCAGCAGGGGCAAGCTGGTGAGGGGTGGGGTGCCGGAGGAGGTCTTCAC
GGCACCCGAGGAGATGGCCAATGTAAAGCTGAGGCTGCCACACATCGCCGAGTTGATCCACCGGCTCAAGCACGAAGACCTGCTCCCATTCGGCCGGATACCGCTGACC
ATCGGCGAGGCGCGGCGGGAGATCGTGGAAACGCTCCGGGAGCGCTGATCAGTACTGCTGTTCAATCACCCTGGCCTTCAGCTTGCCAACGATACTGCCAACAAGGGCC
ATTTGTTCCGGGCTTTCTCCCTCAACCAGCTTGATGTGGCTTGCGTTCACCGGCAGTTGCCCGAAGGTGGGGTCCACGGCCAGCCATTCACCCAGATAGCTTTCCGCCCAG
CAATGGTAGAGAAAACCT

Figure S5. Sequence overlap PCR arrangement. After the concatenation of the various RT-PCR SOPCR amplicons, a single mRNA product was created, confirming the arrangement of *cbiM*, *cbiN*, *cbiO* and *cbiQ* in an operon.

CTCCCGCGGGGCGGGAAAGCTGTTTGCCGGAGATTTGCGCGGAGACAAGATCTGAGGTGAAAAA

ATTCAGCCACGAATAGCACGAAAGTTCACGAATAAGAGCAAAGCGTGAAAGAAAAATATGTGGTTG

TGCTTCGGTTTGACTATTCGTGTTAATTCGTGACATTCGTGGCTAAAAGATTTGAGATTCATA**ATG**AA

cbiM →

AAAAATCCTGTTATTTACAATTTTAATGCAGCTTATTGCTGCCCCTGC**CCATGCCATGCACATCAGTGA**

+1

GGGGCTACTGCCACTGAACTGGGCGCTCTTCTGGTTCATGGCCGCGGCGCCTTTTGTCGCCCTTGG

CGTGCGTCAGCTGAATAACCTGGCAAAAACCGATCTGGCGATGAAACCGCTGGTGGGGCTGATG

GCTGCTGTTGTCTTCATCGTCTCCTGCATGCCCAT

Figure S6. Identification of the +1 transcription start site (+1 TSS) of *cbiM*. The genes *cbiMNQO* are arranged in an operon and the +1 TSS of the *cbi* operon was identified 51 nucleotides downstream of the 5' end of the nucleotide sequence. Identification of only one +1 TSS supported our findings that the genes *cbiMNQO* are arranged in one operon.

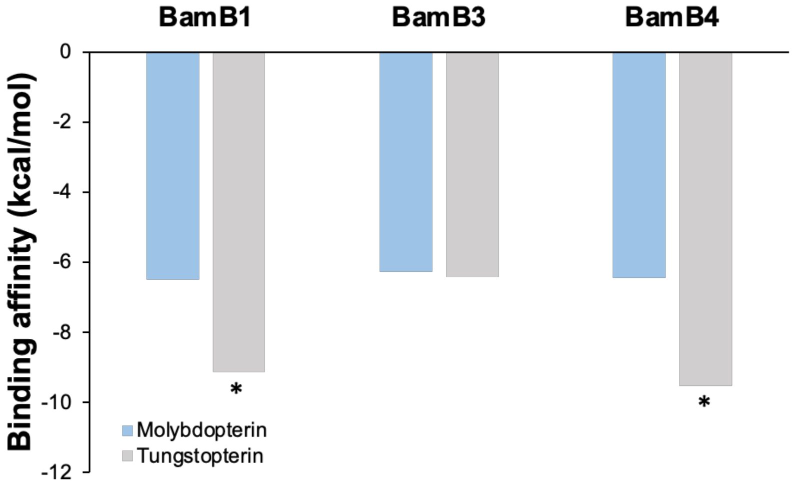

Figure S7. *In-silico* protein-ligand binding affinity prediction of BamB1, BamB3, and BamB4 for molybdo-pterin and tungsto-pterin. Protein-ligand binding affinity predictions were performed in triplicates (*P >0.05, as determined by Student's t-test). Significant difference compared to binding affinity of BamB1 or BamB4 for molybdopterin is indicated by asterisks.

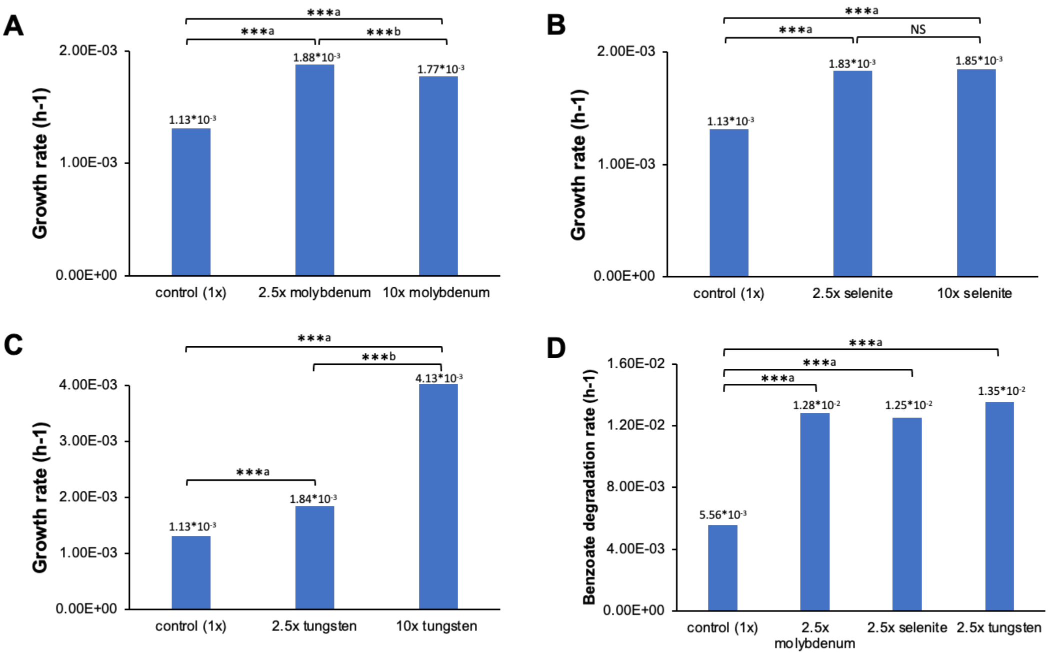

Figure S8. Growth rates and benzoate degradation rates in log phase of anaerobic *G. daltonii* cultures with TE amendments. A, B, C: Growth rates were calculated based on the change of cell density over time during the logarithmic phase of each growth curve displayed in Fig. 5A-C. D: Benzoate degradation rates were calculated based on the change of benzoate concentration over time during the logarithmic phase of each benzoate degradation curve displayed in Fig. 6. The results represent the means ± standard errors of triplicate OD_{600} values or triplicate benzoate concentrations from triplicate cultures (***P >0.0005; as determined by Student's *t*-test). (a) Significant differences compared to growth rate or benzoate degradation rate of control cultures (1×) are indicated by asterisks. (b) Significant differences compared to growth rate of 2.5× cultures are indicated by asterisks.
